# Editorial: New strategies in neuroprotection and neurorepair

**DOI:** 10.3389/fnins.2024.1461195

**Published:** 2024-09-19

**Authors:** Enping Huang, JiaRen Liu, WenBing Ai

**Affiliations:** ^1^Guangzhou Key Laboratory of Forensic Multi-Omics for Precision Identification, School of Forensic Medicine, Southern Medical University, Guangzhou, China; ^2^The Fourth Affiliated Hospital of Harbin Medical University, Harbin, China; ^3^Yiling Hospital of Yichang City, Yichang, China

**Keywords:** neurological disorders, AD, PD, neuroprotection, neurorepair

Neurological disorders, including central nerve injury and peripheral nerve injury, represent a significant challenge to global public health, afflict nearly 50 million people worldwide (Spudich and Nath, 2022; Liang et al., 2021). Central nervous system (CNS) diseases, including neurodegenerative disorders like Alzheimer's disease (AD) (Scheltens et al., 2021; Monfared et al., 2022) and Parkinson's disease (PD) (Bloem et al., 2021; Tolosa et al., 2021), stroke, spinal cord injury (SCI) (Quadri et al., 2020), glioma, depression, and traumatic brain injury (TBI) (Thapa et al., 2021). Nowadays a large number of new technologies and methods are used in the treatment of neurological injuries. Drug therapy (Wang et al., 2020, 2023), operative treatment (Galgano et al., 2017), and physical therapy (Li et al., 2024) are the most common ways to deal with nerve injury. However, many mechanisms of drug therapy, operative treatment, and physical therapy are still unclear. Therefore, we need to develop new theories and methods in neuroprotection and neurorepair.

This Research Topic contains four papers, which focus on therapeutic strategies for AD, PD, glaucoma, and others neurological disorders. The accepted papers are introduced as follows: In “*Flavonoids and fibrate modulate apoE4-induced processing of amyloid precursor protein in neuroblastoma cells*”, Davra and Benzeroual found that fenofibrate, naringenin, and diosmetin treatments decrease Aβ production induced by apoE4, these are potential therapeutic drugs for AD patients. The paper “*Therapeutic value of homeoprotein signaling pathways*”, Nardo and Prochiantz reviewed the role and mechanism of ENGRAILED1, ENGRAILED2 and OTX2 in PD, amyotrophic lateral sclerosis, amblyopia and anxiety-related disorders. In “*Molecular pathways in experimental glaucoma models*”, Bugara et al. reviewed the animal models Of glaucoma and molecular mechanisms involved in the glaucoma development, including TGF-β signaling, neurotrophins, neuroinflammation, chronic oxidative stress, excitotoxicity, ABCA1, Rho-kinase and purinergic signaling pathway. The paper “*Neurotoxic lesions of the anterior claustrum influence cued fear memory in rats*”, Gu et al. found that aCLA but not pCLA was involved in fear memory and extinction in rats.

In conclusion, the “*New strategies in neuroprotection and neurorepair*” Research Topic highlights the most recent and novel therapeutic targets in neuroprotection and neurorepair.
